# An oscillatory pipelining mechanism supporting previewing during visual exploration and reading

**DOI:** 10.1016/j.tics.2021.08.008

**Published:** 2021-09-17

**Authors:** Ole Jensen, Yali Pan, Steven Frisson, Lin Wang

**Affiliations:** 1 Centre for Human Brain Health, School of Psychology, University of Birmingham, U.K; 2 Department of Psychiatry and the Athinoula A. Martinos Center for Biomedical Imaging, Massachusetts General Hospital, Harvard Medical School, Charlestown, Massachusetts 02129, US; 3 Department of Psychology, Tufts University, Medford, Massachusetts 02155, US

**Keywords:** saccades, alpha oscillations, phase coding, preview, visual exploration, reading

## Abstract

Humans have a remarkable ability to efficiently explore visual scenes and text employing eye movements. Humans typically make eye movements (saccades) every ~250ms. Since saccade initiation and execution take 100ms, this leaves only ~150ms to recognize the fixated object (or word), while simultaneously previewing candidates for the next saccade goal. We propose a *pipelining mechanism* where serial processing occurs within a specific brain region, whereas parallel processing occurs across different brain regions. The mechanism is timed by alpha oscillations that coordinate the saccades, visual recognition and previewing in the cortical hierarchy. Consequently, the neuronal mechanism supporting natural vision and saccades must be studied in unison to uncover the brain mechanisms supporting visual exploration and reading.

## The remarkable efficiency of visual exploration and reading

**Figure 1:**
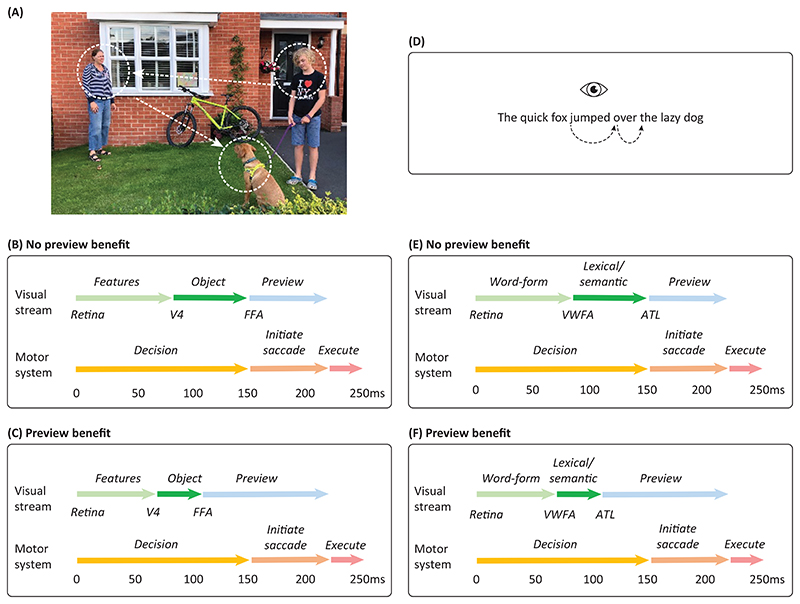
Temporal constraints during visual exploration and reading. A) In this example the participant first fixates on the *boy* and then saccades to the *woman* followed by the *dog.* B) The timing of the visual exploration process. The visual object (the *boy*) arrives at 60 ms in V1 after which visual features are identified at about 85 ms [8]. Electrophysiological evidence suggests that objects can be identified before 150 ms in object selective cortex [16–19]. While this process is going on, the next saccadic decision must be made (*woman* or *dog?*) such that the motor program can commence. It takes about 100 ms to initiate and execute the saccadic motor command [1, 20]. Typically, a saccade is executed about 250 ms after the fixation onset. As both the object identification as well as the saccade decision must be performed with 150 ms, this places serious computational demands on the visual system. C) Previewing by parafoveal processing allows for speeding up visual processing. For instance, when fixating on the *boy* the *woman* can be previewed. When the *woman* then is fixated the recognition can be reduced to about 110 ms [1, 2, 11]. This has two important advantages: 1) it leaves about 40 ms for previewing the next saccade goal (the *dog*) and 2) the preview occurs sufficiently early to impact the next saccade goal (e.g. to skip). D) A sentence is read by fixating on the words sequentially. When fixating on the word *jumped* it must simultaneously be decided on whether to saccade to *over*. E) The timing of the visual reading process. For instance, visual features of the word *over* arrive at 60 ms in V1 after which the word-form is identified in the visual word-form area (VWFA) at ~90 ms. There is electrophysiological evidence suggesting that lexical recognition of the word is done within 150 ms by a network including the mid-fusiform gyrus and possibly the anterior temporal lobe (ATL) [16–19]. While this process is going on, the next saccade decision (*over*) must be made such that the saccadic motor program can be initiated. Both the lexical identification as well as the saccade decision must be performed within 150 ms. F) Parafoveal previewing of a word (e.g. *over*) allows for reducing the lexico-semantic identification upon fixation. As such a previewed fixated word could be recognized at 110 ms. This has an important advantage: it leaves more time for previewing the next word and deciding the next saccade goal before executing the saccade plan. For instance, a decision might be made to skip highly common and/or predictable words.

Our understanding of natural vision presents an intriguing conundrum: How do we manage to efficiently explore visual scenes and text by eye movements given the relatively slow and spatially limited processing capabilities of the human visual system? We saccade every 250 – 300 ms when reading and visually exploring natural scenes. Given that it takes about 100 ms to initiate and execute a saccadic motor program [1, 2], there is only 150 – 200 ms available to process the fixated object or word while in parallel planning the next saccade. Since saccades typically land on informative objects or words [3, 4] (Figure 1), a *parafoveal* **previewing** process is required when exploring and deciding on the next saccade goal. The deployment of pre-saccadic attention has been the topic of many studies [5, 6]. In the context of saccadic exploration, we need to uncover how the visual system can achieve this remarkable computational feat. The fast computation must rely on a highly tuned machinery in which eye movements are coordinated with the visual input [7]. We here suggest a new framework in which visual exploration and reading are supported by a **pipelining** mechanism. This pipelining mechanism is coordinated by neuronal oscillations that serve to organize the visual representations in a temporal code and guide the information through the visual hierarchy.

## The temporal constraints during visual exploration and reading

The recognition of currently fixated objects as well as for deciding on future saccade goals is typically done within 150 ms after fixation onset. This is because saccades are initiated as often as every 250 ms and it takes about 100ms to initiate and execute a saccadic program towards the target [1, 2]. Moreover, it takes about 60 ms for information to travel from the retina to the visual cortex [8], leaving about 90 ms for neocortical processing of the fixated object (Figure 1). Even with such high temporal constraints, it has been shown that it is possible to identify visual objects at the semantic level (“meaning”, such as animacy features) within 150 ms. Multivariate approaches applied to MEG data allow for identifying the *neuronal fingerprint* associated with semantic features [9]. It was found that naturalness and animacy can be decoded from multivariate brain activity at respectively 122 ms and 157 ms after stimulus onset [9]. This timing is consistent with intracranial recordings in monkeys in which object category was decoded within 125 ms in the inferior temporal cortex [10]. Therefore, existing studies suggest that it is possible to identify visual objects at the semantic level (“meaning”) within 150 ms. Because future saccade goals also must be explored and selected within the same time window, this poses a serious computational challenge to the visual system (Figure 1B). Due to the head-start of processing from the parafoveal previewing prior to the saccade, recognition of the fixated object is reduced to about 110 ms [1, 2, 11]. This then leaves ~40 ms more for previewing the upcoming saccade goal (Figure 1C); i.e. it buys time to alter the saccade plan if, for instance, the saccade goal is deemed uninformative. Therefore, the acceleration of visual processing by previewing is likely to be essential for efficient visual exploration.

The temporal constraints during reading are equally tight. After the retinal input has arrived in occipital cortex at 60 ms, the visual word form area (VWFA) is engaged in orthographic processing at about 90 ms [12] (Figure 1D-E). Then follows lexical processing and semantic recognition supported by the left inferior temporal cortex [12]. The lexical processing has been shown to take up to 150ms. Eye-tracking research has demonstrated that fixation times are longer for low- compared to high-frequency words [11, 13] and that the word-frequency effect is present at least within 150 ms as revealed by survival analysis [14, 15] (Figure 1E). Electrophysiological findings also support the notion that lexical identification can take up to 150 ms [16–19]. Since saccade initiation and execution take about 100 ms, there would be almost no time to evaluate the next saccade goal [1, 20] (Figure 1E). Again, previewing will serve to reduce the lexico-semantic recognition possibly to about 110 ms [1, 2, 11], which will leave time to evaluate the next saccade goal (Figure 1F). This allows just enough time to alter the saccade plan if the parafoveal word has already been processed to a sufficient degree that a fixation is no longer necessary.

## How deeply are previewed objects processed?

It is interesting to consider previewing in the context of parafoveal visual acuity. While acuity drops and crowding effects become more prevalent for parafoveal vision (2–5 degrees relative to the current fixation) [21], our eyes still saccade to relevant (and not necessarily salient) parts of visual scenes [3, 4, 22–24]. Using gaze-contingent paradigms occluding the peripheral view, it has been demonstrated that the effective visual span guiding saccades is about 8 degrees [25]. How deeply are previewed objects processed before we saccade to them? In support of previewing at the semantic level, one study demonstrated that search times are faster for objects that are inconsistent with their scene contexts (e.g. *a tube of toothpaste* in the *living room*) as compared to scene-consistent objects (e.g. *a tube of toothpaste* in the *bathroom*) [26]. A recent EEG study investigated fixation-related potentials (FRPs) in response to the fixation on pre-target objects prior to saccading to target objects that were either consistent or inconsistent with the context of the scenes. A larger negative potential at ~300 ms (akin to the N400 type ERP effect) was observed in response to the pre-target when the target object (e.g. *a tube of toothpaste*) was inconsistent with the scene (e.g. *living room*). These findings provide support in favour of parafoveal processing at the semantic level [27].

The perceptual span during reading has also been investigated using gaze-contingent paradigms in which text is occluded to the left and/or right of the gaze. These studies have shown that the visual span extends 14–15 letter spaces (2–3 words) to the right of fixation and 3–4 letters to the left [28] (this effect is reversed in readers of Hebrew who read from right to left [29]). Consistently, it has been shown that occluding the word just to the right of fixation, reduces reading speed by 25–40 ms per word [11]. Interestingly, making the fixated word disappear after 60 ms hardly impacts reading, whereas making the parafoveal word disappear after 60ms, increases reading times substantially [30]. This finding provides strong evidence that fluent reading relies on previewing. How deeply is upcoming parafoveal text previewed? There is strong evidence for previewing at the sub-lexical level (e.g. **orthographic** and **phonological processing**) [31, 32]. Using gaze-contingent boundary paradigms it was shown that fixation times on the target word were reduced after it was primed in the parafovea by an orthographically similar letter string (e.g *sorp* priming *song*) compared to an unrelated condition (*door* priming *song*) (e.g [33, 34]). A similar effect has been found with respect to phonological previewing using homophones [35, 36]. Previewing at the *lexical level* has been investigated using sentences containing target words of low or high lexical frequency. Several eye-tracking studies have found that pre-target fixation times are not modulated by the lexical frequency of the target word, suggesting the absence of lexical previewing [37] (but see [38]). We recently challenged this notion by combining eye-tracking with a **rapid frequency tagging** paradigm. In this paradigm, we subliminally flickered the target words at 60 Hz during natural reading. Using MEG it is then possible to measure the neuronal response at 60 Hz which is specifically associated with the target word. This rapid frequency tagging approach allows for assessing neuronal activity in the visual cortex on a fast time-scale [39]. We found that when readers fixated on the pre-target words, there was a stronger tagging response, as measured by MEG, when the target words were of low compared to high lexical frequency [40], which was not reflected in the pre-target fixation durations. Additionally, faster readers showed stronger parafoveal processing at the neuronal level, indicating that the amount of parafoveal processing affects efficiency in processing. This finding provides novel neural support for previewing at the lexical level, without necessarily impacting behavioral eye-movement measures. Another controversial topic is whether there is previewing at the *semantic level*. This question has been studied using boundary paradigms in which target words changed between semantically related (e.g. *tune* to *song*) or unrelated words (*door* to *song*) when saccading to it. The target word fixation times were not reduced following the semantic parafoveal priming indicating no semantic preview benefits [41]. Surprisingly, when similar studies were conducted using Chinese [42] and German [43], there was support for semantic previewing. In sum, while there is evidence for previewing at the sub-lexical level, there are mixed findings regarding lexical and semantic previewing.

## Pipelining as an alternative to serial or parallel processing

**Figure 2:**
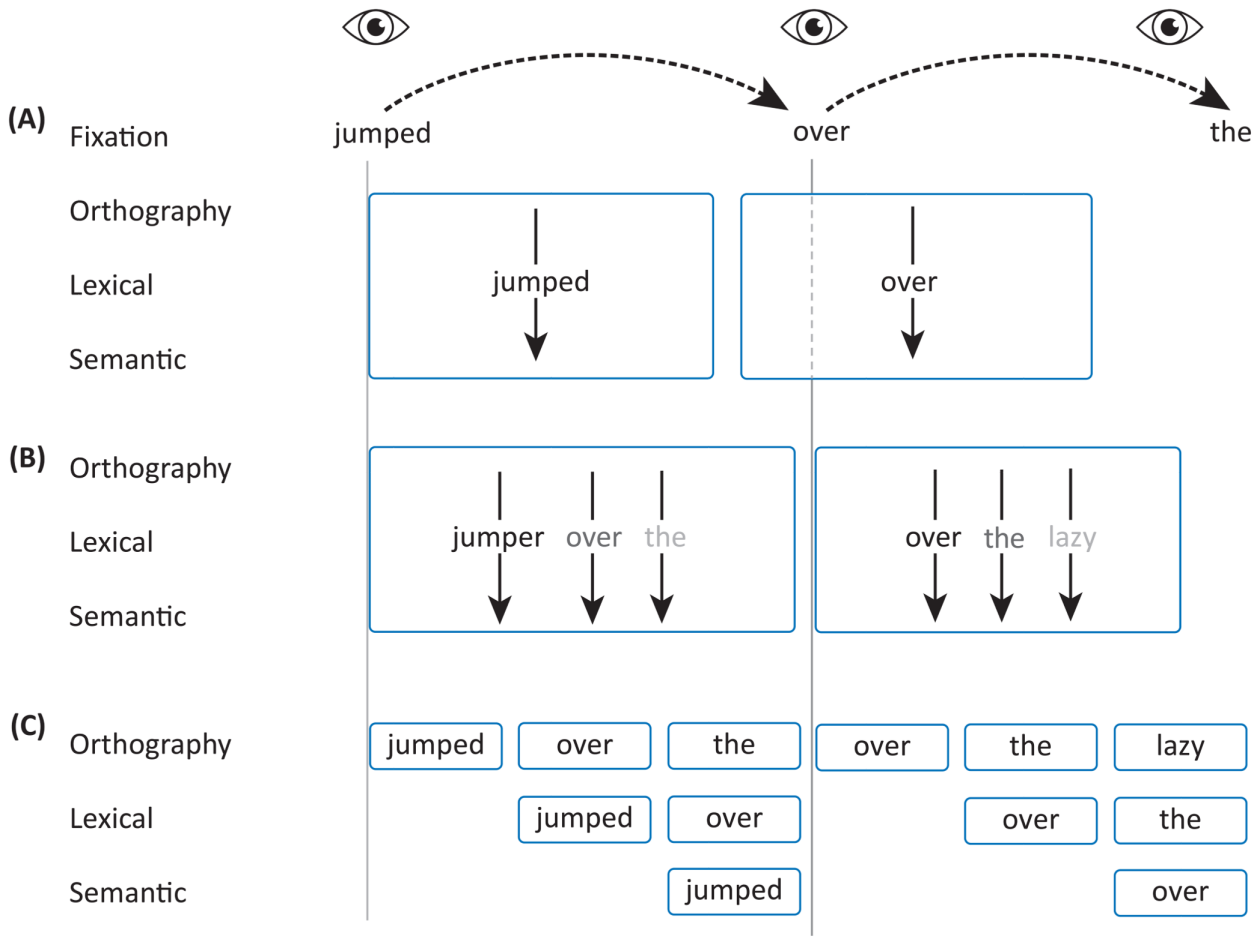
Different models for reading. The eye symbols represent the fixations on the respective words. (a) Serial processing in which each word is processed one at a time. Note that the model still allows for parafoveal on foveal processing but only after the lexical processing of the fixated word is completed. (b) Parallel models for reading suggest that words in the fovea and parafovea are processed simultaneously even at the lexical level. This processing might be graded. (c) We propose that reading is supported by a pipelining mechanism that incorporates elements of both serial and parallel processing. In particular, foveal and parafoveal words (e.g. *jumped* and *over*) are processed simultaneously, however, at different cortical levels in the visual hierarchy.

Different mechanisms have been proposed to account for efficient visual processing, especially during natural reading. It is strongly debated whether visual processing of foveal and parafoveal words during reading is supported by a serial or a parallel mechanism [44]. Proponents of serial mechanisms argue that words are processed lexically one at a time (Fig 2a) [45, 46]. This does not preclude the processing of parafoveal words; however, this is achieved by attention being allocated to parafoveal words after completing the lexical processing of the fixated word (before moving the eyes). These principles are implemented in the E-Z reader model, which can account for a large set of behavioural data [47, 48]. Serial processing is challenged by researchers arguing for a parallel mechanism [49]. The core argument is that words in the fovea and the parafovea are processed simultaneously, albeit in a graded manner (Fig 2b), at the lexical, semantic and even syntactical level [38, 50, 51]. One key argument is based on the phenomenon of word transpositions in reading. It has been shown that readers have trouble classifying a sentence as syntactically incorrect if it can be corrected through the transposition of two words (‘You That Read Wrong Again!‘) [52].

The SWIFT and OB1 models for parallel processing have been developed to account for word recognition in relation to saccade generation [53, 54]. However, the parallel mechanism faces its own challenges. For instance, multiple objects need to be recognized simultaneously, which may result in a bottleneck problem. The bottleneck problem has for instance been studied using crowding paradigms in which the ability to recognize objects is impaired by clutter [55] and it is likely to be a consequence of the hierarchical organization of the temporal lobe. In terms of word recognition and lexical access, the bottleneck problem has been studied using behavioural paradigms and fMRI [56, 57].

We here put forward a pipelining mechanism that incorporates elements from both models while at the same time being compatible with the hierarchical organization of the visual system. The pipelining process is implemented as a multiplexing mechanism, as explained in Fig 2c. After orthographic processing of the fixated word *jumped* is completed, lexical processing is initiated. This then allows for the parafoveal word *over* to be processed simultaneously at the orthographic level. As such, serial processing occurs within a specific brain region, whereas parallel processing occurs across different brain regions. The proposed pipelining scheme does require a precise timing mechanism. As we explain below, neuronal oscillations are ideally suited for coordinating the required temporal organization.

Some components of the E-Z Reader model might be compatible with a pipelining mechanism. For instance, in both models there is a distinction between an early lower-level (orthographic) stage and a later higher-level (semantic) stage of word processing, and both models allow for substantial previewing of upcoming words. However, an important difference is that while previewing or parafoveal processing in the E-Z Reader model is generally delayed until the fixated word has been fully processed (unless the next word is very short, highly frequent, and/or highly predictable), the pipelining mechanism allows for parafoveal processing to start earlier in the cycle, alleviating the bottleneck problem.

## A mechanism supporting natural vision and reading by pipelining coordinated by alpha oscillations

**Figure 3:**
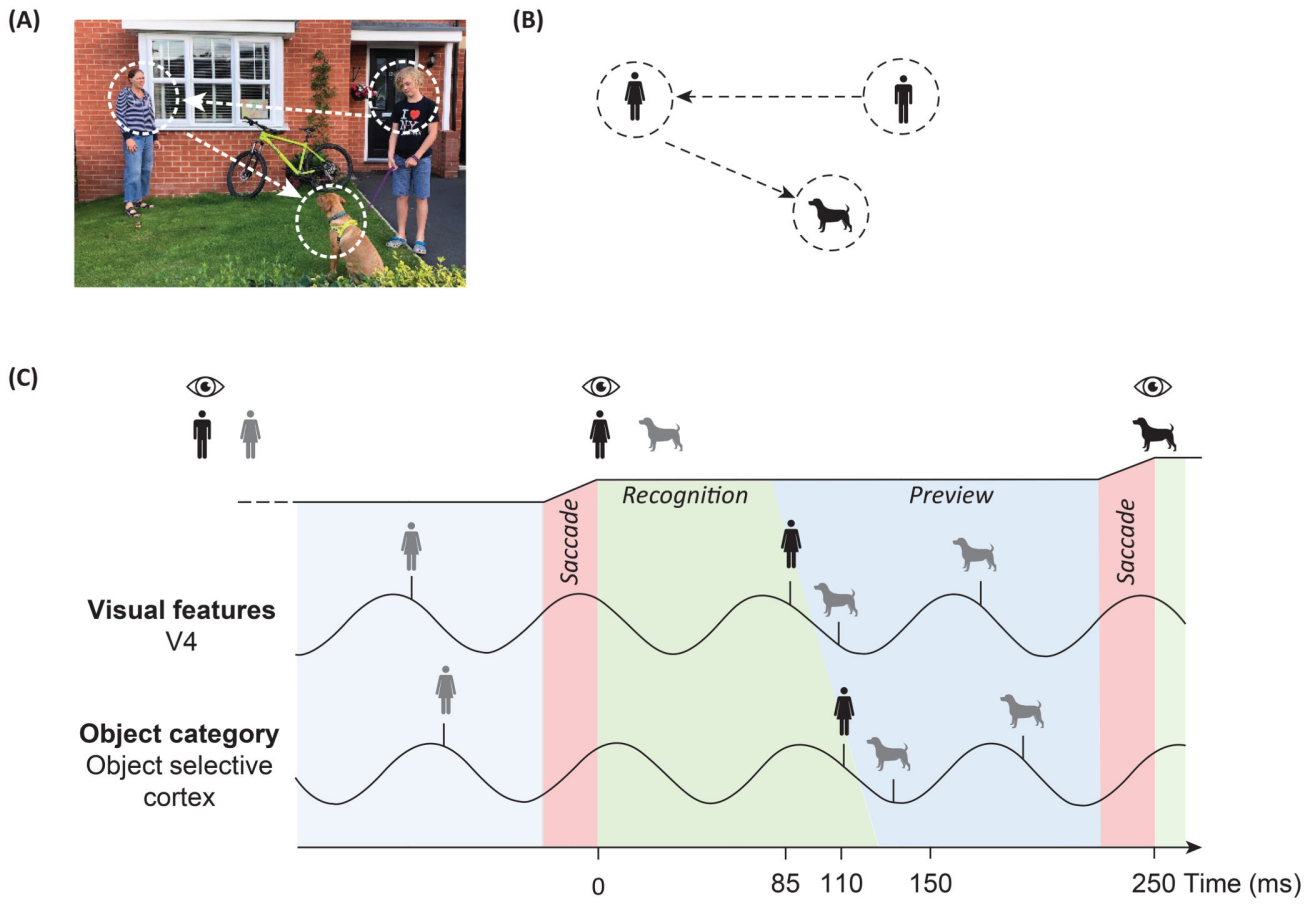
A model for pipelining during visual exploration. A, B) The participant fixates on the *boy* and then saccades to the *woman* and finally to the *dog*. For simplicity, we assume two stages in which simple features (e.g. colour) are identified first (in V4), followed by object category recognition (in object selective cortex in the inferior temporal lobe). C) We hypothesize that the temporal organization supporting the pipelining mechanism is coordinated by oscillations in the alpha band. In this example, 12 Hz alpha oscillations can be considered pulses of inhibition repeated every 83 ms [67, 68]. Note, this is just an example and the mechanism would work with alpha oscillations at slower frequencies as well. At the peak of the alpha cycle, neuronal firing is inhibited. As the inhibition ramps down the most excitable representation will activate and so forth [68]. Consider time point t = 0 ms in which the participant moves the eyes from the *boy* and fixates on the *woman* (the black line on top indicates the horizontal gaze position). We assume that saccades are locked to the phase of the alpha oscillations such that the visual input of the *woman* arrives at the early down-going inhibitory slope of the alpha cycle at about t = 85 ms where simple visual features of the woman engage the visual occipital cortex (e.g. colours in V4). These feature representations are projected to object selective cortex for category identification by 110 ms. This fast category identification is made possible by the preview of the *woman* prior to the saccade which has primed the ‘semantic’ access. Importantly, the pipelining scheme allows the *woman* and the *dog* to be processed in the same cycle in a multiplex manner thus avoiding bottleneck problems. Specifically, the features of the *woman* and the *dog* are processed sequentially in V4 and slightly later the respective object-categories are sequentially processed in object-selective cortex. This scheme allows for a fast decision to be made to either saccade to the *dog* or hold the saccade and preview another object as a potential target.

We propose a pipelining mechanism that can be used to guide efficient visual exploration and reading. We hypothesize that visual exploration relies on a process in which several objects are processed simultaneously at different levels in the cortical hierarchy. Consider Figure 3A in which the viewer fixates on the *woman.* The visual input propagates in the cortical hierarchy in which features of increasing complexity are combined to semantically recognize the object *woman* in inferior temporal (IT) cortex. While the participant fixates on the *woman*, the *dog* is parafoveally previewed as a potential saccade goal. The previewing creates a bottleneck problem due to the hierarchical nature of the visual system as two objects (e.g. *woman* and *dog*) have to be recognized simultaneously. We propose that the bottleneck problem is solved by a pipelining mechanism in which several objects are processed simultaneously but at different levels in the cortical hierarchy (detailed in Figure 2 and Figure 3B). As a consequence, serial processing occurs within a specific brain region, whereas parallel processing occurs across different brain regions. This scheme also serves to coordinate the parafoveal previewing of the *dog* which then will speed up the recognition time when the object eventually is fixated. The pipelining mechanism requires tight temporal coordination. We propose that oscillations in the 8–13 Hz alpha band serve to organize visual presentations according to a **phase code** to support parafoveal previewing and eventually guide the saccadic trajectory. In Figure 3 we have assumed the **alpha oscillations** to be 12 Hz; however, is it possible to scale the mechanism to operate with e.g. 10 Hz alpha oscillations; this will ease the temporal constraints slightly. The saccades must be locked to the phase of the alpha oscillations in order for the processing to be coordinated.

**Figure 4:**
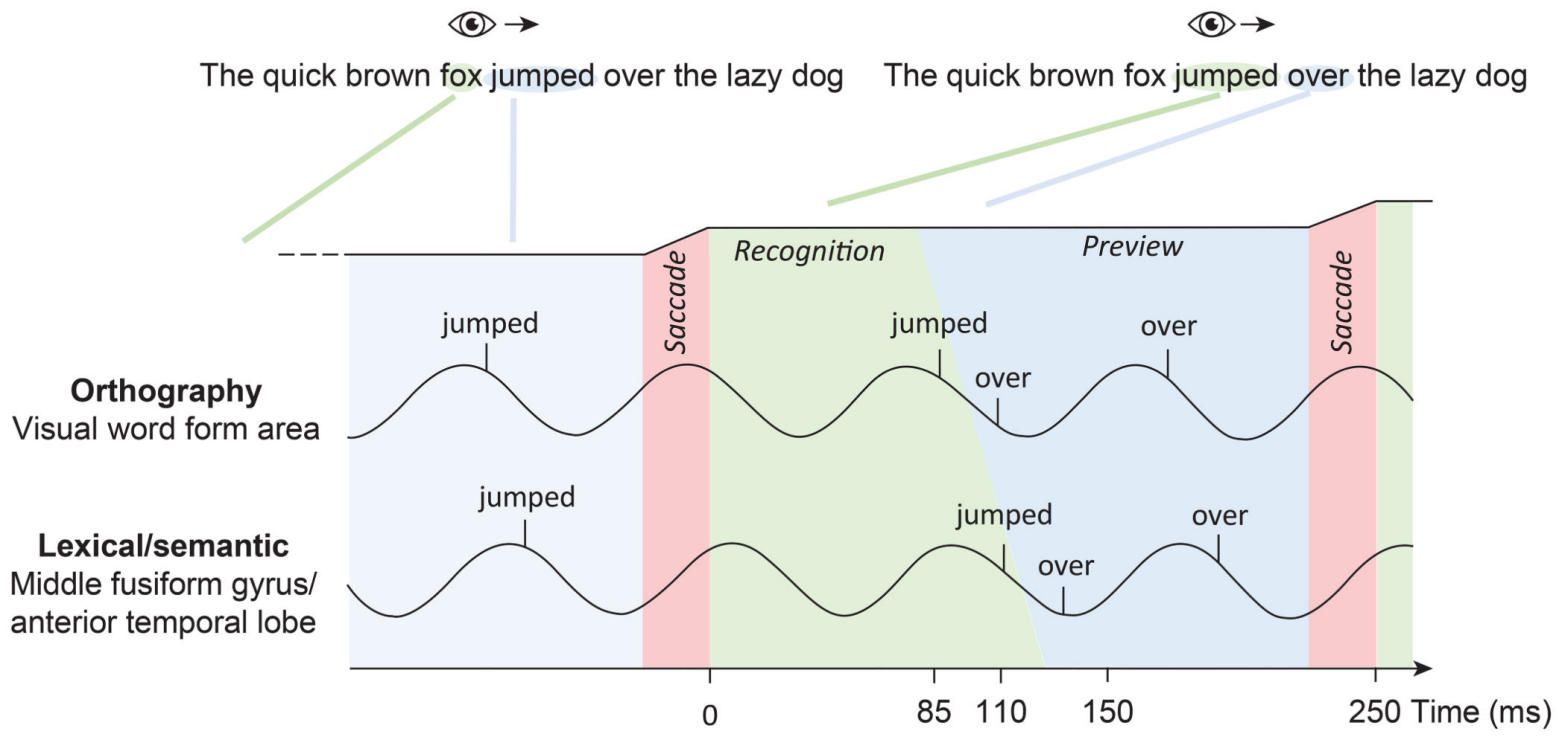
A model for pipelining during natural reading. A) For the sake of simplicity, two stages of word recognition are assumed, namely orthographic identification in the visual word form area (VWFA), followed by lexico-semantic access in an extended network including the middle fusiform gyrus (MFG) and the anterior temporal lobe (ATL). B) We hypothesize that the temporal organization supporting the pipelining mechanism is coordinated by oscillations in the alpha band. The 12 Hz alpha oscillations can be considered pulses of inhibition repeated every 83 ms [67, 68]. Consider time point t = 0 ms in which the subject saccades and fixates on *jumped* (the black line on top indicates the horizontal gaze position). We assume that saccades are locked to the phase of the alpha oscillations such that the visual input of *jumped* arrives at the early down-going inhibitory slope of the alpha cycle at about 85 ms for orthographic feature identification in the VWFA. The orthographic representations propagate to the MFG or ATL for lexico-semantic identification by 110 ms. This fast lexico-semantic process is made possible by the preview of *jumped* prior to the saccade that has primed the lexical processing. Importantly, the pipeline scheme allows both *jumped* and *over* to be lexico-semantically processed in the same cycle but at slightly different points in time, thus avoiding a bottleneck problem in e.g. the MFG and ATL. During the fixation of *over* the word *the* is previewed. Given that the word *the* carries little information and is highly frequent, a decision to skip can be made. However, this is only possible if *over* has been previewed since this will speed up the processing of *over* leaving more time to preview *the*. While this scheme for simplicity only outlines orthographic and lexico-semantic processing, it could be further developed by assigning the lexical and semantic processing to different alpha phases as well as different regions (e.g. respectively the MFG and the ATL). It would also be of great interest to extend the framework to include previewing of two (and not only) words.

A similar mechanism might support natural reading (see Figure 4), with the exception that the saccades are directed to the right. When the word *jumped* is fixated, this allows for *over* to be previewed. As such, different words are processed simultaneously, but at different levels in the hierarchy. As mentioned before, parafoveal previewing results in **lexico-semantic** recognition to be reduced from about 150 ms to 110 ms. This provides extra time for evaluating the next saccade goal and potentially skip a less informative word (e.g. *the*). The pipelining mechanism is organized in a temporal code along the alpha cycle. Importantly, the saccades are locked to the phase of the alpha oscillations in order to organize the processing and the visual input. In short, we argue that efficient visual exploration and reading rely on parafoveal previewing, and the bottleneck problem in the cortical hierarchy is resolved by a pipelining mechanism. The multiplexed processing of fixated and previewed objects is coordinated by alpha oscillations.

A computational mechanism organized as a pipeline requires an intricate temporal organization (Figure 3 and 2). The transfer of representations between levels across the hierarchy as well as the sequential processing of multiple objects within the hierarchy must be coordinated. In the example Figure 3, some of the visual features of the *boy* will propagate from V4 to face-selective areas in IT. Likewise, the face-selective area will process the *boy* slightly earlier than the *woman*. Based on recent findings, we propose that oscillatory coupling in the alpha band serves to coordinate the information transfer between regions [58, 59].

While alpha oscillations for decades were thought to reflect idling or a state of rest [60], it is now evident that they are involved in numerous cognitive processes [61–63]. One key insight is that alpha oscillations are present during continuous visual processing (e.g. [64, 65]). Based on human and animal data, the case has been made that theta and alpha oscillations reflect pulses of inhibition that rhythmically interrupts neuronal firing [63, 66, 67]. These pulses might be implemented by bouts of GABAergic interneuronal input. At the peak of an inhibitory pulse, neurons are prevented from firing. As the inhibition ramps down over the cycle, the most excitable neurons will fire first, then the somewhat less excited neurons and so forth. As such, the pulses of inhibition implement a type of filter, ensuring that neuronal representations are activated sequentially according to excitability [67, 68]. Representations of fixated objects are associated with more neuronal excitability compared to parafoveal representations. This allows foveal representations to overcome the pulsed inhibition earlier and thus activate earlier in the alpha cycle. The sorting of different object representations according to excitability, creates the phase-coded representations which are essential for the pipelining mechanism (Figure 3 and 2). While we have put forward an example with 2 objects in each cycle, the scheme could be extended to 3-4 objects and to include more hierarchical levels. It is also interesting to consider the framework in the light of mechanisms for working memory. We assume that a given word or object is processed over multiple alpha cycles and the respective neuronal representations must be maintained in this interval but not necessarily by sustained firing. Recent research has suggested that working memory can be maintained by activity-silent states involving short-term synaptic plasticity [69–71]. It would be of great interest to investigate if similar mechanisms are at play when maintaining and integrating information over several alpha cycles.

## Predictions and evidence in support of the pipelining mechanisms

The mechanism outlined above rests on several assumptions, some of which are supported by the literature while others need to be empirically tested. One assumption is that information in the visual system is organized according to a phase-code coordinated by neuronal oscillations. A second key assumption is that saccades are locked to the phase of the ongoing oscillations. A third assumption is that the feed-forward flow is coordinated by phase-synchronization between regions in the visual hierarchy in the ventral stream. We will here discuss the empirical support for these assumptions.

## Prediction 1: phase-coding

The proposed mechanism relies on organizing multiple representations in a phase code. Recordings from hippocampal place cells in behaving rats have demonstrated the role of theta oscillations (6 – 12 Hz) in organizing neuronal representations of space. The phenomenon of *theta phase precession* shows that a given place cell fires late in the theta cycle as the rat enters a place field. As the rat advances, the firing precesses to earlier theta phases. This finding is best explained by a mechanism in which a sequence of spatial representations is ‘read out’ within a theta cycle [72, 73]. This phase-coding scheme is consistent with a pipelining mechanism in which different representations along the path are sequentially processed at different theta phases. There is an intriguing link between navigation and visual exploration: both processes require that object representations are anchored to spatial representations in order to represent the environment. It should also be mentioned that despite the different labels, human alpha oscillations and rat theta oscillations overlap in frequency and might support related functions [74].

Phase-coding with respect to neuronal oscillations has also been identified from intracranial recordings in humans performing visual and working memory tasks. For instance, using human intracranial data it was demonstrated that individual working memory representations are represented at different phases of an 8 Hz rhythm [75]. Another intracranial study found that different visual categories were encoded at different phases of the theta oscillations [76]. In non-human primates, work based on intracranial recordings also reports on phase coding in the visual system in various tasks [77–79].

While these reports are encouraging, the phase-coding scheme still needs to be investigated in the context of visual exploration and reading. As outlined in Figures 2 and 3, we predict that alpha-band oscillations coordinate the neuronal processing associated with saccadic visual exploration. As a result, representationally-specific representations for fixated and upcoming saccade goals should be coupled to the phase of the alpha oscillations. This could be tested by MEG or EEG recordings in combination with eye-tracking in humans engaged in visual exploration or reading tasks. It has been shown that different levels of representation associated with upcoming information can be detected using multivariate approaches in MEG and EEG [80, 81]. Therefore, the time-course of the representationally specific activation could be identified by multivariate approaches and then be related to the phase of the ongoing alpha oscillations [82]. As a complementary approach, rapid frequency tagging at different frequencies (50 to 70 Hz) could be used to track several objects and the respective neuronal signals would reflect the neuronal processing of the tagged objects [40]. This could also be achieved using broad-band flicker and temporal response functions (see [83] for methodology). Specifically, we predict that the fixated, as well as the parafoveal object (or word), would become active at different phases of the alpha cycle (see Figure 3C and Figure 4B).

## Prediction 2: alpha oscillations linked to saccades

A second key prediction is that saccades must be linked to the phase of alpha oscillations in order to time the neuronal processing of the visual input. Studies in both humans and animals have found intriguing links between the phase of alpha oscillations and saccades. A study based on both MEG and intracranial human data showed that saccade onsets are locked to the phase of ongoing alpha oscillations when viewing natural images [84]. Importantly, the degree of locking predicted which pictures were later remembered as compared to forgotten. This suggests that visual information impacts memory areas stronger when saccades are coordinated by the phase of alpha oscillations. Using EEG, it was shown that the phase of pre-stimulus alpha oscillations was associated with saccadic response latencies [85]. In another non-human primate study, signals from the V4 receptive field of respectively current and future fixations were coherent in the alpha band around the time of saccades [86]. Not only the allocation of overt attention is related to the alpha phase, so is the allocation of covert attention. A recent study in non-human primates reported on multi-electrode recording in the frontal-eye field that allowed for decoding of the focus of the attentional spotlight. Importantly, the spotlight explored the visual space at a 7-12 Hz alpha rhythm (‘attentional saccades’) [87]. Finally, there is a more tentative link to explore between saccadic suppression and the conjectured pulses of inhibition by alpha oscillations: recording sites in V4 associated with peripheral vision increase in alpha power during saccades [88]. In short, strong evidence is accumulating in support of an intimate connection between saccades and alpha oscillations. Further research is required to establish a link between oscillations and saccades in relation to the proposed phase coding scheme. Specifically, we predict that saccades would be locked to the alpha phase during visual exploration and reading. Possibly a stronger locking would be associated with more efficient visual processing.

While we have not addressed the putative role of microsaccades, this might eventually also be relevant. It has been shown that microsaccades reflect the allocation of spatial attention [89] [90], but surprisingly, microsaccades are mainly leftwards during reading [91]. EEG studies have demonstrated that micro-saccades evoke activity in the alpha band [92] which warrant further investigations.

## Prediction 3: Inter-regional phase-synchronization in the alpha band

A third key requirement for the proposed scheme is that the feed-forward flow of the representations is coordinated by phase-synchronization in the alpha band between regions in the visual hierarchy. This would also support the exchange of phase-coded representations between brain regions [58, 93]. In support, intracranial recordings in non-human primates have shown that synchronization in the alpha band reflects forward communication between V4 and interior temporal areas [94]. Human MEG recordings have demonstrated phase-synchronization in the alpha band between regions in a larger visual hierarchy in participants performing visual attention tasks [95]. We argue that the interregional phase-synchronization in the alpha band could reflect the representational specific feed-forward flow [59, 93]. Other support for temporal coordination by alpha oscillations stems from work demonstrating travelling waves across regions in the alpha band [96], which even are task-dependent [97]. In future work, it would be important to further uncover the phase-relationship between regions in the visual hierarchy during visual exploration and reading tasks. This could be done using MEG or intracranial recordings in patients undergoing presurgical evaluations.

## Concluding remarks

We argue that the visual system must operate in a highly efficient manner to support visual exploration and reading. The core issue is that the fixated object or word must be processed in the same time interval as when the next saccade goal is planned. Given the bottleneck problem in the visual hierarchy [46, 56], we propose that this is achieved by a pipelining mechanism coordinating the processing of current and upcoming visual objects. Importantly, we propose that neuronal oscillations in the alpha band serve to coordinate the pipelining mechanism that is implemented by a phase-coding scheme in which different representations activate sequentially along the phase of the alpha oscillations. Finally, to coordinate the visual input with the neuronal processing, the mechanism also requires that saccades are locked to the phase of the alpha oscillations. The proposed framework has resulted in a set of questions to be addressed in future empirical investigations (see Outstanding Questions).

In sum, we have here presented a novel and testable framework for the neuronal mechanisms supporting visual exploration and reading in relation to saccades. Crucially, neuronal oscillations are required for organizing the visual representation as well as the timing of saccades. Since the proposed mechanism provides a unified account for visual exploration and reading, it also opens the door for future investigations aimed at understanding the neuronal substrate associated with reading and visual disorders.

## Glossary

Alpha oscillations: An 8-13 Hz neuronal rhythm associated with pulsed GABAergic inhibition of neuronal firing.
Lexical processing: The process of retrieving a mental representation of a known word based on orthographic or phonological representations.
Multi-variate decoding: An approach in which the multivariate signals from e.g. EEG/MEG recordings are used to decode the distributed neuronal activity associated with a given feature or object.
Orthographic processing: The process of identifying and combining graphemes to form words.
Phase-coding: A temporal code in which different neuronal representations activate at different phases of ongoing brain oscillations.
Phonological processing: The processing of sounds (e.g. phonemes) of language.
Pipelining: A serial mechanism in which multiple operations are performed in fast succession.
Previewing: The visual processing of parafoveal words or objects.
Rapid frequency tagging: A new approach in which parts of a visual scene are subliminally flickered (‘tagged’) at high frequency (in the 50-70 Hz range). Recording the neuronal response allows isolation of the neuronal activity associated with the tagged object.
Semantics processing: The process that binds together distributed information to form a single concept associated with meaning.

## Highlights

- Humans have a remarkable ability to efficiently acquire information from natural scenes and text by means of saccadic exploration.
- Visual exploration is surprisingly efficient given the temporal and spatial constraints imposed by the visual system. As such, both information from current fixations as well as upcoming parafoveal locations must be processed within a 150 ms time window.
- We propose a novel mechanism in which visual exploration and reading are supported by a pipelining in which serial processing occurs within a specific brain region, whereas parallel processing occurs across different brain regions.
- The timing of the pipelining mechanism is organized by alpha oscillations
- The pipelining mechanism predicts that fixated and parafoveal objects/words are represented at different phases of an alpha cycle.
- Consistent with the mechanism, data in humans and non-human primates point to a link between the timing of saccades and alpha oscillations.

## Outstanding Questions

- How deeply are objects previewed before saccades are made to them? Are they previewed at the semantic level or maybe just in terms of features?
- How deeply are upcoming words previewed during reading before saccades are made to them? Are they primarily previewed at the sub-lexical (e.g. orthographical, phonological, orthographic) or also at the lexico-semantic level?
- What is the role of brain oscillations in visual exploration and reading? Recent studies have found cases where saccades are locked to the phase of alpha oscillations, but how general is this phenomenon in terms of task?
- Are different objects and words represented along the phase of oscillations in the alpha cycle during visual exploration and reading (akin to the coding scheme of place representation organized by theta oscillations observed in exploring rats)? This can be addressed using **multi-variate decoding** applied to MEG and EEG data in order to relate representational specific information to the phase of oscillations in the alpha band. Likewise, *rapid frequency tagging* can be used to investigate the allocation of visual resources already before a saccade in relation to ongoing brain oscillations.
- Is there a link between previewing abilities and reading disorders? For instance, impaired previewing during reading might account for some of the reading deficits observed in some types of developmental and acquired dyslexia. Can our proposed pipelining mechanism account for the impaired previewing?
- Recent evidence suggests that prediction plays an important role in natural vision and language comprehension. How do top-down predictions impact bottom-up parafoveal previewing during reading? Could it be that pre-activated representations and the representations of the previewed objects are encoded at the same phase of the alpha cycle?
